# Synergistic Interaction Effect of *Artemisia cina* n-hexane Extract and *Tagetes lucida* Ethyl Acetate Extract on *Haemonchus Contortus*

**DOI:** 10.1007/s11686-024-00839-6

**Published:** 2024-04-03

**Authors:** Itzel Santiago-Figueroa, Manases González-Cortazar, Julieta Gertrudis Estrada-Flores, Jorge Alfredo Cuéllar-Ordaz, María Eugenia López-Arellano, Francisco Javier González-Reyes, Agustín Olmedo-Juárez, Rosa Isabel Higuera-Piedrahita

**Affiliations:** 1 Facultad de Estudios Superiores Cuautitlán, Universidad Nacional Autónoma de México, Carr. Cuautitlán- Teoloyucan Km 2.5, Col. San Sebastián Xhala, CP 54714 Cuautitlán, México; 2 Centro de Investigación Disciplinaria en Salud Animal e Inocuidad, Agrícolas y Pecuarias (INIFAP), Instituto Nacional de Investigaciones Forestales, Carr. Fed. Cuernavaca-Cuautla No. 8534, CP 62550 Jiutepec, México; 3 Centro de Investigación Biomédica Del Sur, Instituto Mexicano del Seguro Social (IMSS), Argentina No. 1, 62790 Xochitepec, CP México; 4 Instituto de Ciencias Agropecuarias y Rurales (ICAR), Universidad Autónoma del Estado de México (UAEM), Estado de México, Campus UAEM El Cerrillo, El Cerrillo Piedras Blancas, 50090 Toluca, México; 5 Universidad Autónoma Chapingo, Carr. México-Texcoco Km 38.5, C.P. 56230 Chapingo, Estado de México México

**Keywords:** Gastroenteric Nematodes, Small Ruminants, Pharmacology Interaction, Bioactive Plants, Sustainable Anthelmintic

## Abstract

**Purpose:**

We analysed the possible synergistic activity among active extracts from *Artemisia cina* and *Tagetes lucida* combinations on *Haemonchus contortus*, a nematode parasitising sheep.

**Methods:**

The work was carried out in vitro on eggs and infective larvae (L3) of *H. contortus*. The results were analysed with SAS 9.1, applying the ANOVA and Tukey test, and the lethal concentration (LC) values LC50 and LC90 were determined with regression analysis, employing Proc Probit of SAS 9.1. Additionally, the lethal concentration (LC) was calculated with LC_50_ and LC_90_ to determine the synergistic effect.

**Results:**

The results demonstrated a high efficacy of the two plants studied on both nematode eggs and L3 larvae as well as of their combinations. The highest egg hatching inhibition was obtained with a 50/50 combination, and the best larvae mortality was obtained with 25% *A. cina* and 75% *T. lucida* at 10 mg/mL. Additionally, this combination showed a synergistic effect.

**Conclusion:**

The two plant species studied here can be applied as natural anthelmintic alternatives due to their high bioactive effect and synergistic response.

**Supplementary Information:**

The online version contains supplementary material available at 10.1007/s11686-024-00839-6.

## Introduction

Gastroenteric helminths are the cause of major parasitic diseases in grazing ruminants. These disorders cause damage to animal health and welfare and reduce productive performance such as milk production, weight gain, carcass quality and fertility, among others [1]. Haemonchosis is a parasitic disease with high importance in small ruminants. *Haemonchus contortus* feeds on the blood of its host, resulting in anaemia, weight loss, hyperacute infections and sudden death [2]. Due to the rapid development and increase of anthelmintic resistance (AR) in nematode populations worldwide and to diminish the damage caused by these parasites in production systems, the search for pharmacological and non-pharmacological tools is necessary [3, 4]. Within these management strategies, the use of selective deworming with the aid of indicators such as the body condition score [5] and the FAMACHA colour chart [5]. Other practices are focused on population resistance, such as using secondary metabolites from biological organisms, including nematophagous fungi [6] or ruminal bacteria [7].

Plants with secondary metabolites display important anthelmintic activity against gastrointestinal nematodes, including *H. contortus* [8]. Also, various plants have been used in the treatment of anxiety and depression [9], inflammation [10], cancer [9, 11–13], fibromyalgia disorders [14], immunomodulatory control [15], as antioxidants [16] and as pain modulators [17] among others. Flavonoids, as the major compounds of some plants, play a role in the treatment of Parkinson’s disease [18].

The family Asteraceae contains numerous species with benefits for human and animal health. These plants contain several secondary compounds such as terpenoids, flavonoids and other molecules with anticancer, antimalarial and anthelmintic properties [19–21]. Artemisinin is a sesquiterpene lactone obtained from *Artemisia annua* with demonstrated efficacy against malaria, which has been resistant to chloroquine since 1950, along with other properties [19, 21, 22].

*Artemisia cina*, commonly known as Levant wormseed, is a herbaceous plant that has been traditionally used as an anthelmintic agent in many parts of the world, especially in the Mediterranean and Middle Eastern regions. Several studies have shown the efficacy of *A. cina* in controlling various parasitic infections. For instance, a study found that the aqueous and ethanolic extracts of *A. cina* significantly reduced the number of eggs of gastrointestinal nematodes in sheep [23, 24]. Similarly, another study reported that the ethanolic extract of *A. cina* was highly effective in controlling gastrointestinal nematodes in goats [25].

Moreover, *A. cina* has also been found to be effective against other parasitic infections such as malaria, leishmaniasis and schistosomiasis [26, 27]. The active compounds present in *A. cina*, such as santonin, artemisinin and their derivatives, are the main contributors to its anthelmintic activity [28]. A study with an *A. cina* aqueous extract, which was administered to sheep naturally infected with *Moniezia* spp., recorded a 100% efficacy faecal egg count reduction) on at 9th day post-administration [29]. Additionally, an *A. cina* n-hexane extract was assessed against *H. contortus* infective larvae and caused a mortality of 80% at 2 mg/mL. In this same research, the authors administered the *A. cina* n-hexane at 2 mg/kg to gerbils artificially infected with *H. contortus*, resulting in a 100% efficacy [23].

*Tagetes lucida* is another genus (Asteraceae) extensively studied as an anthelmintic since it is rich in some phenolic acids and other metabolites such as coumarins and terpenes [30]. This plant, commonly known as Mexican “Pericón,” is a flowering plant traditionally used for various medicinal purposes, including its cytotoxic activity, which can result in anthelmintic potential [31].

Studies have reported the efficacy of *T. lucida* in controlling parasitic infections. For example, the authors of one study found that the ethanolic extract of *T. lucida* significantly reduced the number of eggs of gastrointestinal nematodes in sheep [32]. Similarly, another study [33] reported that the methanolic extract of *T. lucida* had high anthelmintic activity against the gastrointestinal nematode *H. contortus* in sheep.

Moreover, *T. lucida* has also been effective against other parasitic infections such as malaria and leishmaniasis [34]. The active compounds in *T. lucida*, such as target one, piperine and their derivatives, have been identified as the main contributors to its anthelmintic activity [32].

Combinations of chemical anthelmintics (albendazole and levamisole) with extracts from plants (terpenes) showed an improved nematocidal effect on gastrointestinal nematodes (GIN), suggesting a possible synergistic effect [35]. Combining natural products with chemical anthelmintics could contribute to controlling GIN. Natural plant compounds have a wide range of biological activities, including antiparasitic activity. However, most studies focus on isolated compounds or extracts from single plants, which may not be sufficient to achieve optimal therapeutic effects. Therefore, studying the synergistic effects of different plants could lead to the development of more effective and safer antiparasitic treatments [36].

Several studies have demonstrated the potential synergistic activity of combinations of plant extracts on parasites. For instance, the antileishmanial and antischistisomal activity of four plant extracts and their combinations are promising [26, 28].

A recent review by Athanasiadou [37] summarised the potential of plant synergies for antiparasitic activity. The authors highlight that plant synergies can improve efficacy, reduce toxicity and prevent the development of resistance in parasites. In the present study, using in vitro assays, we evaluated the interaction effects of *A. cina* (*n*-hexane extract) and *T. lucida* (Ethyl acetate extract) against *H. contortus*.

## Materials and Methods

### Plant Material

We purchased 10 kg of fresh pre-flowering leaves and stems of *A. cina* O. Berg ex Poljakov (Asteraceae) from the Hunab laboratory. For identification, a voucher specimen was examined by Dr. Alejandro Torres-Montúfar and deposited at the herbarium of the Facultad de Estudios Superiores Cuautitlán (FES-C) UNAM, México. under voucher no 11,967. The plant was cultivated under specific conditions, including a humidity of 80%, a temperature of 24 °C and a soil pH of 6.3. *Tagetes lucida* aerial parts were harvested in El Cerrillo Piedras Blancas, Toluca Municipality, Mexico State (19.42984 N, -99.669345 W). One plant specimen was deposited in the Herbarium of the Centre for Research in Biodiversity and Conservation, Universidad Autónoma del Estado de Morelos (UAEM-Mexico), under voucher code 33,784.

### *Artemisia* cina Extracts

To obtain the extract, 5 kg of dried *A. cina* leaves and stems were ground and placed in crystal containers with a capacity of 1,000 mL. To extract the compounds, n-hexane was used, and the mixture was kept at room temperature (23–25 °C) for 48 h. The extract was filtered using Whatman No. 4 paper, and the solvent was removed by low-pressure distillation through a rotary evaporator (Heidolph Laborota 4000, Heidolph Instruments, Schwabach, Germany). Finally, the extract was lyophilised and stored at 4 °C for phytochemical and biological assays.

### Tagetes Lucida Extract

To obtain the extract, 5 kg of dried *T. lucida* were macerated in ethyl acetate for 48 h. The extract was filtered using Whatman No. 4 paper, and the solvent was removed by low-pressure distillation through a rotary evaporator (Heidolph Laborota 4000, Heidolph Instruments, Schwabach, Germany).

### Egg Hatching Inhibition (EHI) Test

The EHI test was performed with *H. contortus* eggs obtained from a parasite-free lamb previously infected with the parasite (5,000 infective larvae singular dose, FESC strain, Mexico). The eggs were separated from the faeces (30–50 g) with clean water by sieving through sieves with mesh sizes of 140, 74 and 37 μm and were cleaned in a density gradient with 40% saccharose. The assays were performed in 96-well microtitration plates. Each treatment was tested three times, considering four repetitions per replicate (*n* = 12). The combinations (treatments, *A. cina* extract: *T. lucida* extract) were assigned as follows: (1) Combination 100:00, (2) Combination 0:100, (3) Combination 50:50, (4) Combination 75:25 and (5) Combination 25:75. Each combination was evaluated at 1–5 mg/mL. Additionally, distilled water and Tween 20 (1%) were used as negative controls, and ivermectin (5 mg/mL) was used as positive control. Tween 20 (1%) was used to dilute the evaporated extracts.

An aliquot of 50 µL of an aqueous suspension containing 100 ± 20 *H. contortus* eggs was deposited in each well (experimental unit). Then, 50-µL aliquots of the treatments or controls were deposited, giving a total volume of 100 µL. The plates were incubated at room temperature (25–28 °C) for 48 h, and subsequently, hatching was stopped using 10 µL of Lugol´s solution. The total eggs and larvae (first and second stage) of each well were counted, and the egg hatching inhibition percentage (EHI %) for each treatment was determined using the following formula:

1 $$ EHI\,\% = \left( {\frac{{number\,of\,eggs}}{{number\,of\,eggs + number\,of\,larvae}}} \right)x100 $$

### Larval Mortality Assay

The larvicidal activity of the combination extracts was determined using *H. contortus* infective larvae (L3), which were obtained from a donor animal. The L3 was obtained from faecal cultures by the modified technique of Corticelli and Lai, reported by Reséndiz- González [38]. Larvae were activated with light and washed with 4% sucrose. The assays were performed using 96-well microtitration plates. The treatments were designed as follows: (1) Combination 100:00, (2) Combination 0:100, (3) Combination 50:50 and (4) Combination 75:25 and (5) Combination 25:75. Each combination was tested at 1–10 mg/mL. Additionally, distilled water and Tween 20 (1%) ere used as negative controls, and ivermectin (5 mg/ml) was used as positive control. The plates were incubated at room temperature (18–25 °C) for 24 h. Subsequently, the total larvae, alive or dead, in each well were counted under an optic microscopy Hinotek Lab model L1200, and the mortality percentages were estimated based on the following formula:

2 $$ \begin{gathered} Mortality\,Larvae\,\% = \\ \left( {\frac{{number\,dead\,larvae}}{{numbers\,of\,dead\,larvae + number\,of\,alive\,larvae}}} \right)x100 \\ \end{gathered} $$

### Statistical Analysis

The IEH and mortality percentage data were analysed through ANOVA based on a completely randomised design by the general linear model in the SAS programme. The dependent variables were the combinations and controls. Tukey´s test was performed to identify significant differences among treatments at a 0.05 significance level. The treatments dependent on the concentration were subjected to regression analysis to estimate the lethal concentrations (LC) 50 and 90 using a Probit analysis (SAS®, 2014).

### Synergic Interaction Analysis

To characterise the interactions among the combinations of the extracts, *A. cina* and *T. lucida*, and isobolographic analysis was used following the methodology described by Tallarida et al. [39]. According to this method, the lethal concentrations of 50 and 90 of each extract (assay individual) and their combinations were considered to determine the synergic effect. Likewise, the interaction magnitude of the combinations, the total fraction of LC of the *A. cina* extract and *T. lucida* extract and their combinations were determined according to the following formula:

3 $$ \begin{gathered} TF = \left( {\frac{{LC\,A.cina\,extract\,in\,the\,combination}}{{LC\,A.cina\,extract\,alone}}} \right) \\ + \left( {\frac{{LCT.\,lucida\,extract\,in\,the\,combination}}{{LC\,T.lucida\,extract\,alone}}} \right) \\ \end{gathered}, $$

where:

TF = total fraction, LC = lethal concentration.

Values close to 1 indicate a possible additive effect, and values below 1 indicate a super-additivity or synergistic effect.

## Results

### Ovicidal Activity

The ovicidal activity of both extracts in individual form and their combinations is shown in Table 1. The individual evaluation of *T. lucida* ethyl acetate extract showed the best EHI percentage, close to 90%, at 2.5 mg/mL. At the same time, the *A. cina n*-hexane extract exhibited the lower EHI with the three concentrations used. The combination of 50/50 presented an IEH at almost 100% with all concentrations used (1, 2.5 and 5 mg/mL).

**Table 1:** *Haemonchus contortus* egg hatch inhibition percentages (EIH%) after 48 h exposure to *Artemisia cina* and *Tagetes lucida* extracts and their combinations

		Means of eggs and larvae (L1 or L2) recovered				.
Treatment		Larvae ± s.d		Eggs ± s.d		EHI% ± s.d
*Artemisia cina* (mg/mL)						
1	81.1 ± 8.8		23.1 ± 12.6		21.17 ± 8.2 ^**d**^	
2.5	65.8 ± 13.9		9.3 ± 4.2		7.8 ± 8.3 ^**e**^	
5	66.0 ± 31.8		10.4 ± 13.5		15.2 ± 15.2 ^**de**^	
*Tagetes lucida* (mg/mL)						
1	37.0 ± 10.2		89.0 ± 6.8		71.0 ± 5.6^b^	
2.5	9.2 ± 7.5		72.7 ± 12.2		89.0 ± 8.3 ^**a**^	
5	4.0 ± 3.5		109.1 ± 12.7		96.5 ± 2.9 ^**a**^	
50% *A. cina*/ 50% *T. lucida*						
1	5.0 ± 3.1		109.9 ± 22.9		95.6 ± 2.7 ^**a**^	
2.5	4.4 ± 3.7		64.3 ± 7.8		93.7 ± 5 ^**a**^	
5	2.2 ± 2.8		70.4 ± 14.3		97.3 ± 3.2 ^**a**^	
75% *A cina*/ 25% *T. lucida*						
1	93.8 ± 19.6		13.0 ± 6.7		12.3 ± 6 ^**de**^	
2.5	4.4 ± 3.7		41.9 ± 13.2		90.4 ± 14.6 ^**c**^	
5	6.0 ± 3.1		93.0 ± 6.7		93.3 ± 5.1 ^**a**^	
25% *A cina*/ 75% *T. lucida*						
1	28.0 ± 11.7		72.2 ± 18.6		71.4 ± 13 ^**b**^	
2.5	6.2 ± 4.4		73.0 ± 11.5		92.4 ± 4.7 ^**a**^	
5	4.8 ± 4.2		98.7 ± 19.5		95.7 ± 3.4 ^**a**^	
Ivermectin (5 mg/mL)	0.2 ± 0.7		80.4 ± 14.4		99.7 ± 0.8 ^**a**^	
Distilled water	85.1 ± 14.6		3.9 ± 1.9		4.2 ± 1.6 ^**e**^	
Variation coefficient					11.68	
R^2^					0.96	

* Means with same letter are similar statistically *P* ≤ 0.05, s.d.= standard deviation

### Larval Mortality

A higher larvae mortality was obtained using the individual extracts with a concentration of 10 mg/mL (Table 2). This same response was achieved with the combination of 25/75% of *A. cina* and *T. lucida*, respectively, using 10 mg/mL. The 1 mg/mL concentration resulting in a lower mortality in all treatments except for *T. lucida*. This indicates a dose-response relationship. Ivermectin showed 100% of larvae mortality.

**Table 2:** *Haemonchus contortus* infective larvae mortality after 24 h exposure to *Artemisia cina* and *Tagetes lucida* extracts and their combinations

Treatment	Means died and lives larvae recovered		% Larvae mortality ± s.d.
	Lives ± sd	Died ± sd	
*Artemisia cina* (mg/ml)			
1	72.8 ± 9.5	11.8 ± 3.5	13.6 ± 3^**hi**^
2.5	67 ± 6.2	26.2 ± 7.2	29.2 ± 8.1^**ghi**^
5	66.6 ± 6.5	35.8 ± 7.8	37.3 ± 2.5^**efgh**^
10	10.3 ± 2.3	78.6 ± 12.9	88.2 ± 3.7^**ab**^
*Tagetes lucida* (mg/ml)			
1	69.3 ± 17.6	30.7 ± 7	31.6 ± 5 ^**fgh**^
2.5	45.3 ± 23.6	52.1 ± 15.7	54.4 ± 8.8 ^**def**^
5	25.8 ± 9.0	68.4 ± 15	70.4 ± 8.8^**bcd**^
10	9.3 ± 6.02	56.3 ± 20	85.4 ± 4.7^**ab**^
50% *A. cina/* 50%*T. lucida* (mg/ml)			
1	72.7 ± 13	17.3 ± 3.3	20.1 ± 4.7^**hi**^
2.5	53.2 ± 6.7	61.9 ± 26.6	50.0 ± 10.9^**defg**^
5	29.4 ± 10.1	50.2 ± 14.3	59.8 ± 11.4^**cde**^
10	12.1 ± 7.6	51 ± 23.5	81.2 ± 6.7^**abc**^
75% *A. cina*/25%*T. lucida* (mg/ml)			
1	76 ± 8.9	19.44 ± 3.4	20.0 ± 1.1^**hi**^
2.5	76.5 ± 9.6	32.8 ± 13.4	29.2 ± 6.7^**ghi**^
5	29.7 ± 10.4	45 ± 27.3	55.4 ± 15.8^**def**^
10	25 ± 7	71.7 ± 24.1	72.8 ± 11^**bcd**^
*25% A. cina/ 75% T. lucida* (mg/ml)			
1	61.5 ± 23.4	23.3 ± 11.5	28.5 ± 8.4 ^**ghi**^
2.5	50.8 ± 11.8	50.4 ± 4.7	50.2 ± 8.9^**defg**^
5	18.7 ± 18.3	58.7 ± 32.8	72.8 ± 13.4^**bcd**^
10	10.6 ± 2.1	71.3 ± 6.7	87.1 ± 1.2^**ab**^
Distilled water	87 ± 8.6	5 ± 1	5.8 ± 1.6^**i**^
Ivermectin	0 ± 0	116 ± 11.8	100 ± 0 ^**a**^
Variation coefficient	18.3		
R^2^	0.91		

*Means with same literal are statistical similar. *p* ≤ 0.05

### Lethal Concentration (LC) 50 and 90

The LC_50_ and LC_90_ values calculated for the *A. cina* and *T. lucida* extracts are shown in Table 3. The *T. lucida* extract showed the LC_50_ with s minor concentration, and the *A. cina* extract alone presented the major LC_50_. The LC_90_, by contrast, was obtained for the *A. cina* extract.

**Table 3:** Lethal concentrations (LC) 50 and 90 of *A. cina* and *T. lucida* extracts on larvae 3 of *H. contortus*

Treatment	LC50		IC limit 95% (lower-upper)	Conc. atribuided to A. cina /T.lucida	LC90	IC limit 95% (lower-upper)	Conc. atribuided to A. cina /T. lucida
*T. lucida*		**1.52**	1.2–1.9		**17.78**	12.9–27.9	
*A. cina*		**6.7**	6.2–7.1		**10.6**	9.9–11.7	
50 *A. cina* / 50 *T. lucida*		**3.3**	2.9–3.7	1.65/1.65	**18.4**	14.6–24.9	9.2/9.2
75 *A. cina*/ 25 *T lucida*		**4.36**	3.8-5.0	3.7/1.09	**26.4**	19.9–37.9	19.7/6.6
25 *A. cina* /75 *T lucida*		**2.4**	2.1–2.7	0.6/1.8	**14.07**	11.3–18.5	3.5/10.5

IC = Confidence interval

**Fig. 1:**
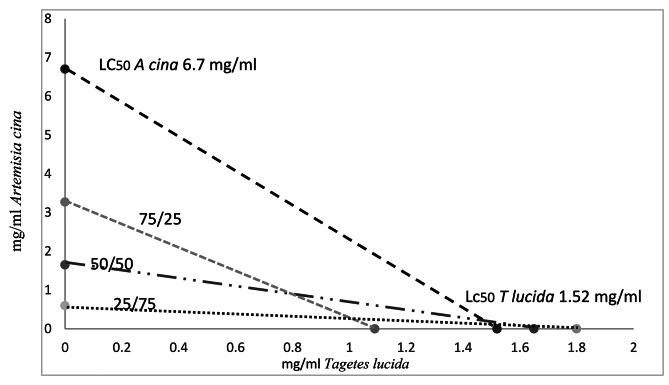
Isobologram of lethal concentration 50 of *Artemisia cina, Tagetes lucida extracts* and some combinations on *H. contortus L3*

The proportion of LC_50_ and LC_90_ attributed for each extract according to the proportion added in the combination is presented in the following table; based on this information, the isobolograms were drawn (Fig. 1).

### Synergistic Activity

The synergistic activity is shown in Fig. 1. According to the estimates for LC_50_, the extracts are more lethal when combined; the isobologram reflects that the concentration needed to cause a 50% mortality of *H. contortus* larvae is lower in all combinations than in the extracts alone; the lines of the three combinations are below the line of the LC_50_ of the extracts alone, suggesting synergistic activity.

The TF (total fraction) for each combination is presented in Table 4. This table shows the effect in all combinations, and both LC_50_ and LC_90_ are sub-additive (antagonism). Moreover, for the 25:75 *A. cina*: *T. lucida* combination showed a synergistic effect was obtained.

**Table 4:** Total Fraction (FT) extracts from *Artemisia cina* and *Tagetes lucida* combinations according to the proportions used, for CL_50_ and CL_90_

Combination	Proportion	Concentration lethal		Total fraction	Effect
		*A. cina*	*T. lucida*		
*A. cina: T. lucida*_CL50_	50:50	1.65	1.65	1.3	Sub-additive (antagonic)
	75:25	3.27	1.09	1.2	Sub-additive (antagonic)
	25:75	0.6	1.8	1.3	Sub-additive (antagonic)
*A. cina*: *T. lucida*_CL90_	50:50	9.2	9.2	1.3	Sub-additive (antagonic)
	75:25	19.2	6.6	2.2	Sub-additive (antagonic)
	25:75	3.5	10.5	0.9	Synergistic

## Discussion

*Artemisia cina n*-hexane extract contains lignans with potent antiparasitic activity, such as isoguaiacine and norisoguaiacine [40]. The major compounds identified in *T. lucida* are caffeic acid, ferulic acid, coumaric acid and chlorogenic acid [41]. The synergistic effects of plant extracts have gained considerable attention as in alternative treatments of parasitic infections in animals [42]. Plants contain several bioactive compounds, and their combination may enhance their efficacy as antiparasitic agents. Several studies have reported the benefits of using plant extracts in combination to treat parasitic infections in animals. For instance, Khanolkar [42] demonstrated that the combination of *Piper longum* and *Tinospora cordifolia* extracts showed a synergistic effect against gastrointestinal nematodes in sheep. Similarly, Bhosale et al. [43] found that the combination of extracts from various plants, including *Achyranthes aspera*, *Vernonia amygdalina* and *Lantana camara*, had a synergistic effect against gastrointestinal nematodes in goats.

The synergistic effects of plant extracts may be due to their ability to target different stages of the parasite’s life cycle or to other physiological mechanisms. In addition, combining compounds may lead to increased bioavailability and better absorption, resulting in a higher efficacy [44]. Moreover, the use of plant extracts in combination may help to reduce the development of resistance to the treatment as it is less likely for parasites to develop resistance to multiple compounds simultaneously [45].

Synergistic effects of metabolites from plants of the genus *Artemisia* and secondary metabolites from other plants have been previously evaluated and identified. Essential oils of *A. herba alba* and *Lavandula angustifolia* have an antibacterial effect against *Escherichia coli*, *Pseudomonas aeruginosa* and *Staphylococcus aureus* at 20 µL/mL when used individually and at 0.5 and 0.1 µL/mL when used in combination, indicating synergistic activity [46]. The activity was calculated with volume concentrations [46]. In addition, a trial conducted on antibiotic-resistant *Staphylococcus aureus* strains with a combination of several flavonoids from *A. rupestris* and norfloxacin showed a TF of less than 0.5 (synergistic effect) [47]. In another study, two of the significant methoxylated compounds from the dichloromethane fraction of the *T. lucida* plant showed a possible synergistic effect of coumarins and flavonoids against fungi [48]. These findings may explain the results obtained in the present work.

*Artemisia cina* is active against *H. contortus*. In a previous study, *A. cina* 30 CH (centesimal Hahnemannians) administered to sheep naturally infected with gastrointestinal helminths reduced egg counts per gram (69%) and inhibited egg hatching by 100% in vitro [49]. In another study, *n*-hexane extract was active against *H. contortus* larvae [23]. Based on these reports, this type of extract was selected in the present study. Recently, for this extract, two lignan compounds responsible for the activity against *H. contortus* have been identified, namely 3’-demethoxy-6-O-demethylisoguaiacine and norisoguaiacine [40]. However, the anthelmintic effect observed for the *n*-hexane extract was more significant than that found in the present study as it resulted in a larval mortality of 80% at 4 mg/mL [40], whereas in our study, a similar effect was found using 10 mg/mL.

The aerial parts of the plant *T. lucida* contain several metabolites, mainly coumarins, flavonoids and terpenes [30], with anti-inflammatory effects [30] as well as antimicrobial effects at 450 mg/mL using petroleum ether and chloroform extracts on bacteria such as *Escherichia coli*, *Enterobacter alcalifaciens* and *Staphylococcus aureus* [50]. In other studies, its effect against fungi such as *Aspergillus niger* and *Fusarium sporotrichum* has also been demonstrated, obtaining the highest activity with the methanol/dichloromethane fraction and identifying coumarins as the compounds with the most significant effect [48]. In other studies, *T. lucida* essential oil was applied against the nematode *Nacobbus aberrant* in tomato plants, achieving an 85% inhibition of gall formation when applied at 10 mg/mL [4, 51].

## Conclusions

*Artemisia cina* and *Tagetes lucida* extracts can act synergistically against the nematode *Haemonchus contortus* due to their high anthelmintic activity and similar active compounds.

### Electronic Supplementary Material

Below is the link to the electronic supplementary material.

Supplementary Material 1

Supplementary Material 2

## Data Availability

The data supporting these findings are available from the corresponding author upon reasonable request.
