# Supplementary material for: Synergistic Interaction Effect of Artemisia cina n-hexane Extract and Tagetes lucida Ethyl Acetate Extract on Haemonchus Contortus
Source: Acta Parasitol. 2024 Apr 3;69(2):1132–40. doi: 10.1007/s11686-024-00839-6 (PMC11182837; doi:10.1007/s11686-024-00839-6)
Supplement: Supplementary file 1 — Supplementary Material 1 [file 11686_2024_839_MOESM1_ESM.pdf]

UNAM
CUAUTITLÁN

UNIVERSIDAD NACIONAL AUTÓNOMA DE MÉXICO
FACULTAD DE ESTUDIOS SUPERIORES CUAUTITLÁN

Mexico, October 16, 2023

Professor
Daniel Młocicki
Editor-in-chief
Acta parasitologica

Dear Editor

Please find the manuscript entitled "**Synergistic interaction of *Artemisia cina* n-hexanic extract and *Tagetes lucida* ethyl acetate extract on *Haemonchus contortus***" for revision by Acta Parasitologica. This study aimed was to evaluate the interaction of plants Artemisia cina (n-hexane extract) and *Tagetes lucida* (Ethyl acetate extract) on *in vitro* assays effect against *Haemonchus contortus* for identify synergistic activity among these extracts.

The manuscript is an original contribution, has not been published elsewhere in substantially the same form, that it is not currently under consideration elsewhere.

We would like being very pleased if after revision, this manuscript is considered as suitable for publication in your prestigious journal.

Yours sincerely,

Ph.D. Rosa Isabel Higuera-Piedrahita

Profesora de Carrera Asociado C
