# Supplementary material for: Synergistic Interaction Effect of Artemisia cina n-hexane Extract and Tagetes lucida Ethyl Acetate Extract on Haemonchus Contortus
Source: Acta Parasitol. 2024 Apr 3;69(2):1132–40. doi: 10.1007/s11686-024-00839-6 (PMC11182837; doi:10.1007/s11686-024-00839-6)
Supplement: Supplementary file 2 — Supplementary Material 2 [file 11686_2024_839_MOESM2_ESM.pdf]

Cuautitlán Izcalli, 6 de marzo de 2024

Dear

Daniel Młocicki

Editor in Chief

Acta Parasitologica

Dear Editor,

Here, we have prepared a list of the comments and suggestions made by the Reviewer, and we have included the individual answers to every comment. Likewise, the corrections suggested by the Reviewer are being addressed in the new manuscript version. We hope this new version fills the standard quality required by your prestigious journal.

Reviewer #3: At first, the presented manuscript about synergistical effect of two plant extracts needs extensive English revision. Furthermore, I have some major comments to the manuscript. In general, the manuscript needs to be proofread as in this version, a mixture of *A. cina* and *Artemisia cina* is throughout the manuscript, similarly the T. lucida and *H. contortus*. These needs to be in italics, at the present form, it seems that it was submitted in haste.

Authors: The manuscript was sent to proofreading service for style review. The voucher is attached, and the changes made are highlighted in yellow.
The *A. cina* and *T. lucida* were uniformed: italics.

Reviewer #3: The abstract states that HPLC characterization was made, but in results there is only note that it was done in another papers 28) and Díaz-Mediana 2021 (not in the references), this needs to be erased from the abstract.

Authors: The sentence was erased from the abstract, and the reference Díaz-Medina was added to the references.

Reviewer #3: I don´t think that term "anthelmintic non pharmacological" is correct.

Authors: The term was erased and changed by “as natural anthelmintic alternative” (line 35)

Reviewer #3: The introduction is too long, several reports could be brought together and appropriately referenced. Eg. L.95-98 together with l.99-101.

Authors: The lines 95 to 101 were combined and rewrited.

Reviewer #3: Line 56- there are certainly more studies about anthelmintic activity of plant secondary metabolites than ref [9], better some review on this topic should be referenced (e.g. Natural Products Are a Promising Source for Anthelmintic Drug Discovery - PMC (nih.gov))

Authors: The reference was added

Reviewer #3: The methodological section should be more extensive.
Eg. L. 128 Include „parasite free" lambs if this was the case.

Authors: The sentence parasite-free was added

Reviewer #3: What solvent was used to dilute the evaporated extracts?

Authors: Tween 20 (1%) was used to dilute the evaluated extracts. Line 130.

Reviewer #3: was the carrier used as a negative control?

Authors: The Tween 20 (1%) was used as a negative control. Line 129.

Reviewer #3: Are the concentration 1-5 mg-ml in the final solution?

Authors: The concentrations evaluated were 1 to 5 mg/ml. Line 128. The final volume was 100 µL

Reviewer #3: What was the concentration of the solvent there?

Authors: 50% of the extract was diluted with Tween 20 and subsequently brought to the final volume with distilled water (final volume 100 µL—line 128 to 130.

Reviewer #3: l. 135 IVM twice „ml"
Authors: The sentence was corrected

Reviewer #3: l. 147 At least brief description of L3 preparation should be included.

Authors: The protocol was added. Line 141 – 142

Reviewer #3: l. 153 "dead or alive larvae", what was the difference? Was it motility? Or larvae shape? Usually larval migration test is performed using L3s.

Authors: The difference between larvae was motility. The authors review motility and classify it as alive or dead.

Reviewer #3: In general ivermectin has limited ovicidal activity, here the concentration used 1-5mg/ml are far too high. But also for larval viability. E.g. very new study of Encalada -Mena 2024 (Exp. Parasitol) used for larval migration assay concentrations up to 25μM which corresponds to 0.022 mg/ml.

Authors: The ivermectin dose was taken from protocol reported by Reséndiz-González et al., 2022.

Reviewer #3: In results as mention before the first section about the composition of extracts should be moved to the discussion since it was reported by other papers.

Authors: The paragraph was moved to the discussion. Line 203 - 208

Reviewer #3: l.189 "mortality larval" ?

Authors: The mortality larval was evaluated by alive or death of larvae. We assessed it in a microtitration plate after 24 hours of exposition.

Reviewer #3: In Table 1, There is little „b" in the fourth line also above the mean. I did not check all the numbers, but just by quick overlooked, there are two number exactly the same in the 75:25 (13.0 ±6.7) check i fit is correct and in the same section the 55.5 EHI cannot be correct.

Authors: There was an incorrect date in the table. The value was corrected.

Reviewer #3: I don´t think it is correct to estimate LC90 when the highest concentration of both extracts used (10 mg/ml) did not reach this level of mortality. And similarly for the combinations. Therefore I think the LC90 isobolgram (Figure 2)should be deleted.

Authors: The isobologram LC90 was deleted.

Reviewer #3: In the Discussion, there are some parts unclear.
l.231 The paper mentioned used different concentration units probably, ul/ml, this need to be explained.

Authors: The explanation was included Line 222

Reviewer #3: L241 mentioned 69% per gram…of what, this is also unclear. The papers mentioned in the discussion needs to be understandable as they are in this manuscript without the need to look back to original papers.

Authors: The sentence was rewritten. Line 229 – 231

Reviewer #3: The last sentence of the discussion, l.256 is exaggerated, these studies only support the idea of anthelmintic effect, but don´t „explain" it whatsoever.

Authors: The sentence was erased. Line and we 245

Reviewer #4: The manuscript is interesting.

Authors can read these articles and include them in the introduction to highlight the role of medicinal plants in other disorders

Fernández J, Silván B, Entrialgo-Cadierno R, Villar CJ, Capasso R, Uranga JA, Lombó F, Abalo R. Antiproliferative and palliative activity of flavonoids in colorectal cancer. Biomed Pharmacother. 2021 Nov;143:112241.

Ferrarini EG, Paes RS, Baldasso GM, de Assis PM, Gouvêa MC, Cicco P, Raposo NRB, Capasso R, Moreira ELG, Dutra RC. Broad-spectrum cannabis oil ameliorates reserpine-induced fibromyalgia model in mice. Biomed Pharmacother. 2022 Oct;154:113552.

Şahin TÖ, Yılmaz B, Yeşilyurt N, Cicia D, Szymanowska A, Amero P, Ağagündüz D, Capasso R. Recent insights into the nutritional immunomodulation of cancer-related microRNAs. Phytother Res. 2023 Oct;37(10):4375-4397

Mitra S, Das R, Emran TB, Labib RK, Noor-E-Tabassum, Islam F, Sharma R, Ahmad I, Nainu F, Chidambaram K, Alhumaydhi FA, Chandran D, Capasso R, Wilairatana P. Diallyl Disulfide: A Bioactive Garlic Compound with Anticancer Potential. Front Pharmacol. 2022 Aug 22;13:943967.

Ferreira PMP, Almeida AAC, Conceição MLP, Pessoa ODL, Marques LGA, Capasso R, Pessoa C. Cordia oncocalyx and oncocalyxones: From the phytochemistry to the anticancer action and therapeutic benefits against chronic diseases. Fitoterapia. 2023 Sep;169:105624.

Goni O, Khan MF, Rahman MM, Hasan MZ, Kader FB, Sazzad N, Sakib MA, Romano B, Haque MA, Capasso R. Pharmacological insights on the antidepressant, anxiolytic and aphrodisiac potentials of Aglaonema hookerianum Schott. J Ethnopharmacol. 2021 Mar 25;268:113664

Khan MF, Kader FB, Arman M, Ahmed S, Lyzu C, Sakib SA, Tanzil SM, Zim AFMIU, Imran MAS, Venneri T, Romano B, Haque MA, Capasso R. Pharmacological insights and prediction of lead bioactive isolates of Dita bark through experimental and computer-aided mechanism. Biomed Pharmacother. 2020 Nov;131:110774.

Cerqua I, Neukirch K, Terlizzi M, Granato E, Caiazzo E, Cicala C, Ialenti A, Capasso R, Werz O, Sorrentino R, Seraphin D, Helesbeux JJ, Cirino G, Koeberle A, Roviezzo F, Rossi A. A vitamin E long-chain metabolite and the inspired drug candidate α-amplexichromanol relieve asthma features in an experimental model of allergen sensitization. Pharmacol Res. 2022 Jul;181:106250.

Açar Y, Ağagündüz D, De Cicco P, Capasso R. Flavonoids: Their putative neurologic roles, epigenetic changes, and gut microbiota alterations in

Parkinson's disease. Biomed Pharmacother. 2023 Dec;168:115788

D'Avino D, Cerqua I, Ullah H, Spinelli M, Di Matteo R, Granato E, Capasso R, Maruccio L, Ialenti A, Daglia M, Roviezzo F, Rossi A. Beneficial Effects of *Astragalus membranaceus* (Fisch.) Bunge Extract in Controlling Inflammatory Response and Preventing Asthma Features. Int J Mol Sci. 2023 Jun 30;24(13):10954..

Kocyigit E, Kocaadam-Bozkurt B, Bozkurt O, Ağagündüz D, Capasso R. Plant Toxic Proteins: Their Biological Activities, Mechanism of Action and Removal Strategies. Toxins (Basel). 2023 May 24;15(6):356.

Uddin Chy MN, Adnan M, Chowdhury MR, Pagano E, Kamal ATMM, Oh KK, Cho DH, Capasso R. Central and peripheral pain intervention by Ophiorrhizarugosa leaves: Potential underlying mechanisms and insight into the role of pain modulators. J Ethnopharmacol. 2021 Aug 10;276:114182.

Authors: The papers were read and included in the manuscript. Line 53 – 63.

Reviewer #4: Do the authors think that the microbiota may be involved in improving the therapeutic effect?

The authors can read these articles where the role of the microbiota in various pathologies is highlighted.

Ağagündüz D, Cocozza E, Cemali Ö, Bayazıt AD, Nanì MF, Cerqua I, Morgillo F, Saygılı SK, Berni Canani R, Amero P and Capasso R (2023), Understanding the role of the gut microbiome in gastrointestinal cancer: A review. Front. Pharmacol. 14:1130562

Ağagündüz D, Çelik E, Cemali Ö, Bingöl FG, Özenir Ç, Özoğul F, Capasso R. Probiotics, Live Biotherapeutic Products (LBPs), and Gut-Brain Axis Related Psychological Conditions: Implications for Research and Dietetics. Probiotics Antimicrob Proteins. 2023 Aug;15(4):1014-1031.

Ağagündüz D, Kocaadam-Bozkurt B, Bozkurt O, Sharma H, Esposito R, Özoğul F, Capasso R. Microbiota alteration and modulation in Alzheimer's disease by gerobiotics: The gut-health axis for a good mind. Biomed Pharmacother. 2022 Sep;153:113430.

Authors: The authors definitely consider that there is a relationship between the clinical picture that produces a disease and the immunological status given by the immune system and the digestive system. Of course, the microbiota can enhance the effect of specific metabolites and also control or accentuate the resilience phenomenon of animals.
In the present work, we cannot evaluate the effect of the microbiota since our approaches are in vitro. Of course, research continues to assess the compounds we have here in the definitive host, such as sheep.

Reviewer #4: Do the authors have information on the safety of the preparation?

Authors: The safety of *A. cina* was probed from 2021 to 2023; the paper is in press in Scientific Reports Journal. The authors evaluated the therapeutic and four higher doses following the OECD guides for toxicity evaluations. Our results showed no alterations in biochemical parameters in blood at therapeutic doses and no histopathological changes in the liver, brain, kidney, lung, and heart.

Reviewer #4: In the Discussion, the Authors should highlight the possible clinical significance of their findings

Authors: The relevance of the present study was added to the discussion. Line 209 – 210.

The proofreading service is attached below:

Proofreading complete: Santiago Figueroa estilo (ref. no. 202402-2902449)

Charlotte Brown <p376rs@gmail.com>
Mar 05/03/2024 12:10 PM
Para:ROSA ISABEL HIGUERA PIEDRAHITA <rhiguera05@comunidad.unam.mx>

2 archivos adjuntos (199 KB)
Santiago Figueroa estilo_clean_version.docx; Santiago Figueroa estilo_tracked_version.docx;

Proof-Reading-Service.com
PhD theses, journal papers, books and other professional documents

Proof-Reading-Service.com Ltd, Devonshire Business Centre, Works Road, Letchworth Garden City, Hertfordshire, SG6 1GJ, United Kingdom
Office phone: +44(0)20 31 500 431
E-mail: enquiries@proof-reading-service.com
Internet: http://www.proof-reading-service.com
VAT registration number: 911 4788 21
Company registration number: 8391405

Dear Rosa Higuera,

We have completed the proofreading you asked for. Please find attached two versions of each document. One is the tracked version, showing all the changes our proofreader has made. You can use the tracking function of Word to accept or reject each change individually. The second is the clean version, which you can use if you do not wish to review the changes we have made. Please note there may be some comments from the proofreader in both versions.

If the clean version still shows corrections please follow the following steps: Press Ctrl+Shift+E and you should see in the task bar a new menu, click on the drop down menu and select 'Final'.

When you are submitting or resubmitting your article to a scientific or academic journal, remember to inform the journal editor in your covering letter that your paper has been professionally proofread. We will be delighted to provide you with verification that your article has been proofread by PRS, so please request a certificate to accompany your paper, especially if the journal editor has already indicated a need for professional proofreading.

If you have further questions about the proofreading, feel free to get in touch.

Thank you for using our service this time. We would be very happy to provide you with further services in the future!

We have recently been experiencing some problems with emails and attachments from some University and business accounts. If you have not heard from us within 2 hours during normal business hours after you have sent your work it is possible that your email has not been received. Please send it from a webmail account such as Hotmail, Yahoo or GMAIL.

Yours sincerely,

Charlotte Brown

Yours sincerely,

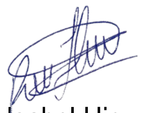

Rosa Isabel Higuera-Piedrahita

Corresponding Author
